# Transcriptomic Analysis of the Antiviral Responses in Ovine Type II Alveolar Epithelial Cells During Early Stage of Bluetongue Virus Infection

**DOI:** 10.3390/ani16020243

**Published:** 2026-01-13

**Authors:** Yunyi Chen, Nijing Lei, Zhenghao Ye, Shaohua Pu, Shimei Luo, Xianping Ma, Shaoyu Yang, Guanghua Wang, Huaijie Jia, Huashan Yi

**Affiliations:** 1 College of Veterinary Medicine, Southwest University, Chongqing 402460, China; 2 Guangxi Key Laboratory of Marine Environmental Disaster Processes and Ecological Protection Technology, Beibu Gulf University, Qinzhou 535011, China; 3 Qinghai Academy of Animal Sciences and Veterinary Medicine, Qinghai University, Xining 810016, China; 4 State Key Laboratory for Animal Disease Control and Prevention, Lanzhou Veterinary Research Institute, Chinese Academy of Agricultural Sciences, Lanzhou 730046, China

**Keywords:** bluetongue virus, ovine type II alveolar epithelial cells, IPA analysis, antiviral immunity

## Abstract

Bluetongue virus (BTV) infects various ruminant species, posing significant threats to animal health and causing substantial economic losses to the livestock industry. Ovine type II alveolar epithelial cells (OAECIIs) play crucial roles in maintaining pulmonary structural integrity and modulating immune responses. Their dysfunction is closely associated with lung disease pathogenesis, making them important therapeutic targets. However, OAECIIs’ immunoregulatory functions and early response mechanisms during BTV infection remain unclear. To address this, we analyzed transcriptomic changes in OAECIIs following BTV-1 infection. RNA-seq revealed 1047 and 852 differentially expressed genes (DEGs) at 8 and 12 h post-infection (hpi), respectively, compared to uninfected controls. Bioinformatics analysis showed significant upregulation of nucleic acid-sensing receptors, interferon-stimulating factors, inflammatory mediators, and cytokines during early infection, mediated primarily through type I interferon signaling, TNF signaling, and cytosolic DNA-sensing pathways. We identified MAD5, ZNFX1, cGAS, OAS, PKR and ZBP1 as key pattern recognition receptors in OAECIIs during BTV infection. The IFN-β, MX1/2, RSAD2 and PLSCR1 pathways mediated antiviral responses, while IL-15, CXCL10, CCL2 triggered inflammatory responses, collectively causing structural alterations through AQP1/9 and tight junction protein modulation. These findings provide critical insights into early antiviral mechanisms and cellular structural changes in OAECIIs during BTV infection, establishing a foundation for understanding pneumonia pathogenesis and developing targeted BTV therapies.

## Simple Summary

This study systematically characterized the innate immune and inflammatory pathways activated during early BTV infection through transcriptome sequencing of BTV-infected ovine type II alveolar epithelial cells at 8 and 12 h post-infection (hpi), integrated with bioinformatics analysis and qPCR validation. Furthermore, we demonstrate that early BTV infection disrupts cellular structural integrity. These findings not only enhance our understanding of the host’s early immune response to BTV-1, but also provide critical insights into virus–host interactions and the molecular mechanisms underlying bluetongue disease pathogenesis, thereby establishing a solid experimental basis for the development of antiviral intervention strategies.

## 1. Introduction

Bluetongue (BT) is an arboviral disease caused by the bluetongue virus (BTV), a double-stranded RNA (dsRNA) virus belonging to the Orbivirus genus within the Reoviridae family. BTV typically causes mild clinical signs or subclinical infections in wild ruminants, cattle, and goats, while sheep tend to suffer the highest mortalities (average 2–30%), which are primarily transmitted by blood-feeding midges of the Culicoides spp. midges [1]. The pathogenesis of BT is characterized by capillary leakage, leading to pulmonary edema, pleural effusion, and pericardial effusion. These pathophysiological changes contribute to the high mortality observed in infected ruminants [2]. Recent advances in RNA sequencing (RNA-seq) technology have enabled comprehensive profiling of host cell responses to BTV infection across species, including sheep, bovine, and murine cell models, thereby providing critical insights into the molecular mechanisms underlying BTV pathogenesis [3,4,5]. Given the lungs’ role as the primary shock organs in ruminants and BTV predilection for replicating within the endothelial cells of infected tissue [6], studies have demonstrated that BTV infection induces the activation of pulmonary microvascular endothelial cells (PMECs). This activation triggers the release of inflammatory mediators, cytokines, microvascular injury, and expedited disease progression. Additionally, our laboratory has recently demonstrated that BTV invades ovine lung microvascular endothelial cells (OLMECs), where it modulates apoptosis, cell migration, and immune activation via the extracellular matrix (ECM) signaling pathway [7,8,9].

The alveolar-capillary barrier, also known as the blood-gas barrier (BGB), is a highly specialized structure in the lungs, composed of tightly interconnected alveolar epithelial cells (AECs), microvascular endothelial cells (MVECs) and the ECM that separates them [10]. The BGB acts as a physical barrier that separates the bloodstream from the alveolar gas environment. It selectively permits the passage of gas molecules while maintaining bidirectional gas exchange with minimal resistance. With its ultra-thin structure of only 0.2–0.3 μm, the BGB minimizes the diffusion distance for oxygen (O_2_) and carbon dioxide (CO_2_), thereby enabling rapid and highly efficient gas exchange. However, this structural feature paradoxically renders the BGB highly vulnerable to viral infections. Its thinness and exposed endothelial surface make it an ideal target for invasion by respiratory and bloodborne pathogens, which may accelerate disease progression [11]. Among these, AECIIs play a pivotal role in alveolar homeostasis through multiple critical functions. These include that active alveolar fluid clearance via Na^+^/K^+^-ATPase-mediated ion transport to regulate pulmonary edema, surfactant secretion to maintain alveolar stability and reduce surface tension, cytokine release (e.g., IL-6, IL-8) to modulate monocyte recruitment and immune activation. Additionally, AECIIs produce antimicrobial peptide (e.g., β-Defensins, Cathelicidins) and inflammatory mediators synthesis to regulating local immunity, thereby suppressing pathogen proliferation during infection. Collectively, these functions establish AECIIs as central immunomodulatory sentinels within the alveolar epithelium, bridging innate immune defense with tissue repair and homeostasis [12,13,14]. Following infection with respiratory influenza viruses (IV) or SARS-CoV-2, AECIIs secrete interferons, inflammatory cytokines, and chemokines, exhibiting dual antiviral and pro-inflammatory properties. However, excessive and prolonged inflammatory responses indicate significant dysregulation of innate immunity, culminating in diffuse alveolar damage, pulmonary edema, and other pathological changes [15,16]. Notably, recent studies demonstrate that IV induced type I interferons (IFN-I) fail to effectively suppress viral replication and instead disrupt the structural integrity of neonatal AECIIs, further exacerbating pulmonary pathology [17].

BTV is highly sensitive to interferon (IFN), and the IFN-mediated innate immune response serves as the host’s primary and most critical defense against viral infection [18]. However, BTV has evolved multifaced strategies to antagonize IFN signaling through multiple pathways, thereby enhancing viral replication and promoting efficient viral release [19,20,21]. To date, most studies on BTV-host immune interactions have centered on post-infection periods exceeding 12 h post-infection (>12 hpi). However, BTV achieves endosomal escape and genome release within 2–4 hpi, and progeny virions are detectable in mammalian cells (e.g., BHK-21) as early as 6–8 hpi [3,22,23,24]. This rapid viral lifecycle creates a critical gap in our understanding of the mechanisms governing early immune responses (<12 hpi), which remains poorly characterized and limits insights into BTV pathogenesis. Based on the aforementioned observations, we hypothesize that BTV enters OLMECs via the bloodstream, subsequently damaging to ovine alveolar epithelial cells (OAECs), particularly ovine alveolar type II epithelial cells (OAECIIs), and ultimately culminating in pulmonary dysfunction in sheep. To elucidate the molecular mechanisms underpinning BTV’s early immune evasion and pathogenesis, we performed transcriptomic profiling of OAECIIs at 8 and 12 hpi. This study aimed to identify key regulatory pathways governing the virus–host interactions during this critical early phase of infection.

## 2. Materials and Methods

### 2.1. Ethical Considerations

All procedures were carried out in strict compliance with institutional biosafety guidelines. This study was based on archived viral samples collected during routine surveillance activities and did not require any additional animal experiments.

### 2.2. Cells and Viruses

Primary ovine type II alveolar epithelial cells (OAECIIs) were obtained from iCell Cybiocon (SHE-iCell-a005, Shanghai, China). The cells were cultured in the iCell Primary Epithelial Cell Culture System (PriMed-iCell-001, iCell Cybiocon, Shanghai, China), supplemented with 5% fetal bovine serum (FBS; Gibco, Grand Island, NE, USA) and 1% penicillin-streptomycin (P1400, Solarbio, Beijing, China), under a humidified atmosphere of 37 °C with 5% CO_2_. The viral strain used in this study was YNDH/103/2013 (BTV-1, GenBank accession numbers: PQ168272–PQ168281). It has been stored at −80 °C under strict biosafety containment conditions.

### 2.3. Cell Culture and BTV Infection

OAECIIs were seeded at a density of 1 × 10^6^ cells per well in 6-well plates and cultured in 2 mL of complete medium (iCell Primary Epithelial Cell Culture System) per well. Upon reaching 80–90% confluence, cells in the experimental group were infected with BTV-1 strain YNDH/103/2013 at an MOI of 1, whereas the control group was treated with virus-free medium. Following incubation at 37 °C for 1 h, the inoculum was removed, and each well was replenished with 2 mL of fresh complete medium for continued culture. All infection experiments were independently repeated three times (*n* = 3) to ensure adequate statistical power. At 8 h and 12 h post-infection, cells in each well were lysed by the direct addition of 1 mL TRIzol™ Reagent (Invitrogen, Carlsbad, CA, USA). The resulting lysates were transferred to Majorbio Bio-Pharm Technology Co., Ltd. (Shanghai, China) for RNA sequencing analysis.

### 2.4. RNA Extraction and Qualification

Total RNA was extracted from the TRIzol solution (Section 2.3) using chloroform (Sigma-Aldrich, Taufkirchen, Germany) and isopropanol (Sigma-Aldrich, Taufkirchen, Germany). RNA quality was assessed using an Agilent 5300 Bioanalyser (Agilent Technologies, Inc, Santa Clara, CA, USA) and quantified using a NanoDrop 2000 spectrophotometer (Thermo Fisher Scientific, Inc, Waltham, MA, USA). High-quality RNA samples were selected for transcriptomic library construction. A total of 9 RNA samples were subjected to transcriptome sequencing, generating an average of more than 6.27 Gb of clean data per sample, with a Q30 base percentage exceeding 95.34%. The clean reads from each sample were aligned against the Oar_rambouillet_v1.0 sheep reference genome (“Ovis_aries_rambouillet-Ensembl Genome Browser 113”, version unspecified), achieving alignment rates ranging from 96.5% to 96.76%.

### 2.5. Library Construction and Sequencing

RNA purification, reverse transcription, library construction and sequencing were performed at Shanghai Majorbio Bio-pharm Biotechnology Co., Ltd. (Shanghai, China), according to the manufacturer’s instructions. Total RNA (1 μg) was used to construct the OAECIIs RNA-seq transcriptome library using an Illumina^®^ Stranded mRNA Prep, Ligation Kit (San Diego, CA, USA). Poly(A)-selected mRNA, isolated using oligo (dT) beads, was fragmented with a fragmentation buffer and reverse transcribed into cDNA using a SuperScript double-stranded cDNA synthesis kit (Invitrogen, CA, USA) with random hexamer primers. The synthesized cDNA was end-repaired, phosphorylated, and adapter-ligated according to the library construction protocol. Target cDNA fragments of approximately 300 bp were isolated using 2% Low Range Ultra Agarose gel electrophoresis and PCR-amplified using Phusion DNA polymerase (NEB) for 15 cycles. The prepared libraries were quantified using Qubit 4.0 and sequenced on a NovaSeq X Plus platform (PE150, version 1.2.2) with the NovaSeq Reagent Kit. Raw paired-end reads were trimmed and quality-controlled using fastp software (version 0.23.2) [25] with default parameters. Clean reads were aligned to the Ovis aries reference genome (Oar_rambouillet_v1.0) using HISAT2 (version: 2.1.0) in orientation mode [26]. The mapped reads from each sample were assembled using StringTie (version 2.2.2) [27] in a reference-based approach to reconstruct transcripts.

### 2.6. Bioinformatics Analyses

Differentially expressed genes (DEGs) were identified using the DESeq2 (version 1.42.0) tool at various time points post BTV infection, with selection criteria set at fold change (|log_2_FC| ≥ 1) and false detection rate (FDR) < 0.05. Venn diagrams were used to analyze unique and overlapping DEGs of BTV-infected cells across different time points. Genetic Ontology (GO) and Kyoto Encyclopedia of Genes and Genomes (KEGG) enrichment analyses were performed on the DEGs to identify significantly enriched biological processes, cellular components, molecular function, and metabolic pathways associated with the host response to BTV-1.

### 2.7. IPA Analysis of Differentially Expressed Genes

The Ingenuity Pathway Analysis (IPA, http://www.ingenuity.com, accessed on 10 April 2025) (Qiagen, Redwood City, CA, USA) software was employed to perform a systematic analysis of the functional pathways associated with DEGs. Pathways exhibiting statistically significant differences (*p*-value < 0.05) were identified and further evaluated. Additionally, Upstream Regulator Analysis and Causal Network Analysis were conducted on DEGs at 8 hpi and 12 hpi to elucidate potential regulatory mechanisms.

### 2.8. Protein–Protein Interaction (PPI) Analysis for Key Genes in OAECIIs

To identify key functional genes in OAECIIs at two time points following BTV-1 infection, PPI networks were constructed using an online analysis tool STRING (Version 11.5). Network diagrams detailing interactions between DEGs were generated, and tabular data of functionally related target–target protein interactions were exported. Further analysis of the PPI network was conducted using Cytoscape (Version 3.10.2). The CytoHubba (version 0.1) plugin was employed to calculate degree values and determine the top 10 hub genes. Meanwhile, the MCODE (version 2.0.3) plugin was utilized for clustering analysis of DEGs. Additionally, the CytoNCA plugin was used to visualize the importance distribution of genes within the PPI network.

### 2.9. Real-Time Quantitative PCR

Reverse-transcriptase quantitative PCR (RT-qPCR) was performed on an ABI QuantStudio 3 system (Applied Biosystems, Foster, CA, USA). After collecting control and BTV-infected OAECIIs, cells were washed three times with 1× PBS to remove residual medium and contaminants. Total RNA was extracted with TRIzolTM reagent and reverse-transcribed into cDNA. HiScriptIII RT SuperMix (+gDNA wiper) and ChamQ Universal SYBR qPCR Master Mix (Vazyme Biomedical Technology, Nanjing, China) were used for RT- qPCR. The primer sequences are listed in Table 1. All primers were synthesized by BGI Genomics Co., Ltd. (Shenzhen, China). The qPCR protocol: initial denaturation at 95 °C for 2 min, 40 cycles of amplification at 95 °C for 5 s and 60 °C for 30 s, and melting curve analysis at 95 °C for 5 s and 60 °C for 5 s. Gene expression levels were normalized to β-actin, the internal control.

### 2.10. Statistical Analysis

Relative gene expression levels were calculated using the 2-ΔΔCt method. Statistical analysis was performed with GraphPad Prism 7.0 (GraphPad Software, San Diego, CA, USA). Data are presented as mean ± SD from at least three independent experiments. Significance was determined by Student’s *t*-test: * *p* < 0.05, ** *p* < 0.01, *** *p* < 0.001, ns (not significant).

## 3. Results

### 3.1. Viral Infection and Verification

**Figure 1:**
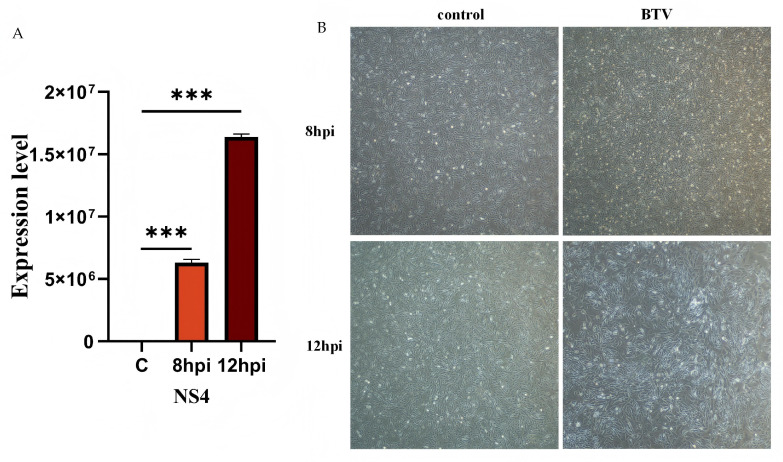
**Validation of BTV-1 infection in OAECIIs at 0, 8, 12 hpi.** (**A**) RT-qPCR was performed to verify the successful infection of BTV-1. (**B**) The morphological alterations in OAECIIs following BTV-1 infection were observed. *** denotes *p* < 0.001.

OAECIIs were infected with BTV-1 at MOI = 1. Viral replication was confirmed by RT-qPCR quantification of viral RNA at 8 and 12 hpi (Figure 1). NS4 expression was significantly upregulated at both time points (*p* < 0.001; Figure 1A). Microscopic observation revealed obvious cytopathic effects (CPE) in the infected group, but not in the control (Figure 1B).

### 3.2. Inter-Sample Correlation and Gene Overlap Analysis

**Figure 2:**
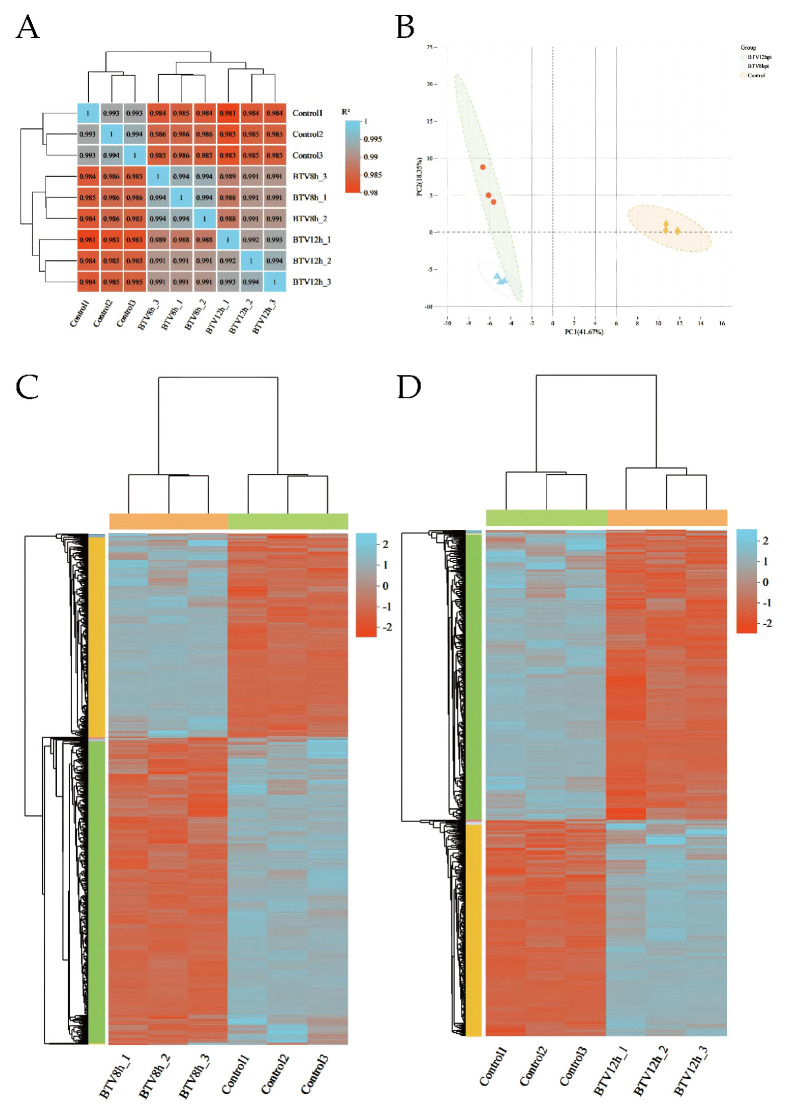
Sample correlation heatmap (**A**) and principal component analysis (PCA) plot (**B**). Hierarchical clustering heatmap of BTV-1-infected samples at 8 hpi (**C**) and 12 hpi (**D**). The dendrogram shows sample clustering; shorter branch distances indicate higher similarity in gene expression. Color intensity reflects normalized log10(FPKM + 1) values, with orange indicating high expression and blue low expression.

The inter-sample correlation heatmap (Figure 2A) and principal components analysis (PCA) plot (Figure 2B) show clear separation between infected groups (BTV-8 hpi, BTV-12 hpi) and the control, with high consistency among replicates, confirming good reproducibility. This supports the reliability of downstream analyses. Clustering of DEGs in BTV-1-infected OAECIIs revealed distinct temporal expression patterns, with marked differences in gene expression between infected and control groups (Figure 2C,D).

### 3.3. Differential Gene Expression Analysis

**Figure 3:**
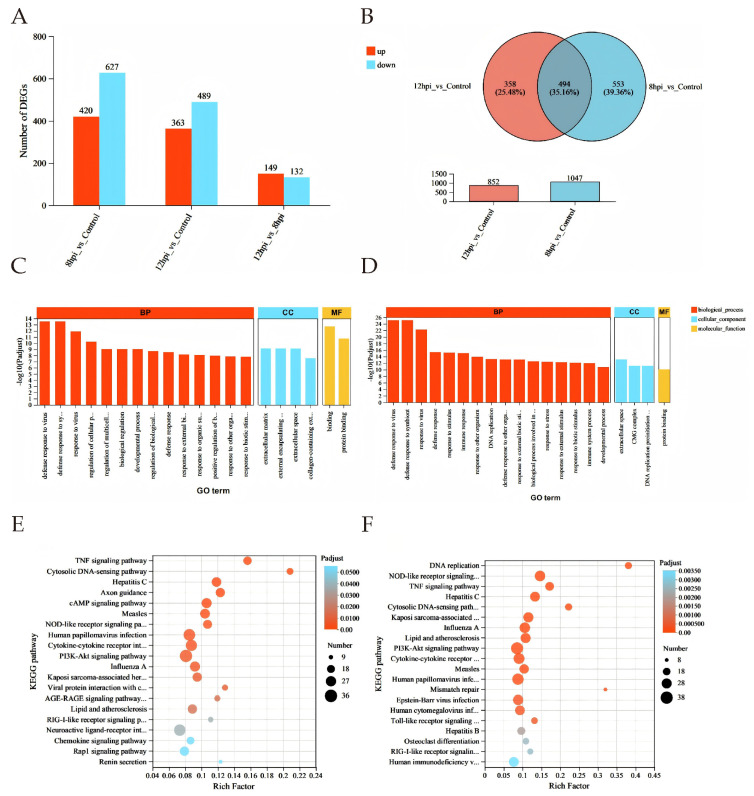
**Differential gene expression analysis.** (**A**) Bar chart of DEGs at 8 and 12 hpi. (**B**) Venn diagram showing DEGs at both time points; red: upregulated, blue: downregulated. (**C**,**D**) **GO enrichment analysis** at 8 hpi (**C**) and 12 hpi (**D**). DEGs in biological process (BP, red), cellular component (CC, blue), and molecular function (MF, yellow). *X*-axis: GO terms; *Y*-axis: –log10(P-adjust). (**E**,**F**) **Top 20 KEGG pathways** at 8 hpi (**E**) and 12 hpi (**F**). *X*-axis: Rich factor; bubble size: number of genes; color: adjusted *p*-value range.

Differential expression analysis revealed 420 upregulated and 627 downregulated genes in BTV-1-infected OAECIIs at 8 hpi, and 363 upregulated and 489 downregulated genes at 12 hpi compared to controls (Figure 3A). A total of 494 common DEGs were identified across both time points, with significantly reduced expression levels at 12 hpi versus 8 hpi (Figure 3B). To characterize the biological functions of DEGs, GO enrichment analysis identified the top 20 significant terms (*p* < 0.05), showing enrichment in defense response to virus (GO:0051607), molecular binding (GO:0005488), and extracellular matrix organization (GO:0101212) at both time points (Figure 3C,D; Tables S1 and S2). KEGG analysis revealed immune-related pathways such as TNF (map04668), cytosolic DNA-sensing (map04623), and NOD-like receptor signaling (map04621). Enrichment of Hepatitis C (map05160) and Influenza A (map05164) pathways suggests BTV-1 shares infection mechanisms with these viruses. At 12 hpi, significant enrichment in DNA replication (map03030) and mismatch repair (map03430) indicates BTV-1 may exploit host replication machinery (Figure 3E,F; Tables S3 and S4).

### 3.4. IPA Analysis

**Figure 4:**
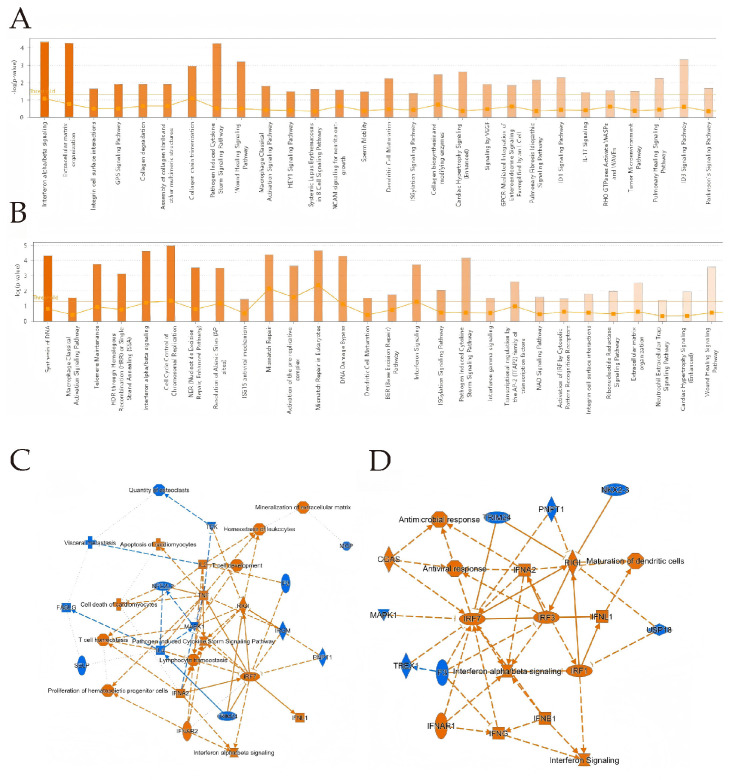
**IPA analysis** was conducted on DEGs at 8 hpi (**A**) and 12 hpi (**B**). The bar chart shows the top 20 pathways with the highest enrichment of DEGs in IPA canonical pathways, sorted by -Log10 (*p*-value). Orange indicates activation, blue indicates inhibition, and color intensity reflects the degree. Ratio represents the proportion of DEGs to total pathway genes. (**C**,**D**) **Downstream regulatory effects** of DEGs. In (**C**,**D**), orange ellipses indicate activation, blue ellipses indicate inhibition; orange lines represent activation, blue lines represent inhibition, double lines denote direct interaction, and dashed lines signify indirect interaction.

IPA analysis showed that in BTV-1-infected OAECIIs, DEGs at 8 hpi were enriched in interferon alpha/beta signaling, extracellular matrix organization, and pathogen-induced cytokine storm pathways. At 12 hpi, DEGs were enriched in DNA synthesis, ISG15 antiviral mechanism, and cell cycle control of chromosomal replication (Figure 4A,B). IPA uses the Activation z-score to predict upstream regulator activity. The TNF z-score decreased from 2.051 to 1.43 between 8 and 12 hpi, indicating reduced activation. Key regulators STAT1 and TWIST1 play central roles in viral infection signaling. STAT1 activates antiviral genes downstream of interferon signaling. TWIST1 may regulate immune responses and infection progression by modulating gene expression (Table 2 and Table 3). IRF3 and IRF7, key factors in the interferon pathway, are activated at both time points (Figure 4C,D), indicating sustained antiviral responses. At 12 hpi, an “Antiviral response” node links CGAS and RIG-I, expanding the regulatory network.

### 3.5. PPI Delineates Critical Genes in OAECIIs That Are Implicated in BTV Infection

**Figure 5:**
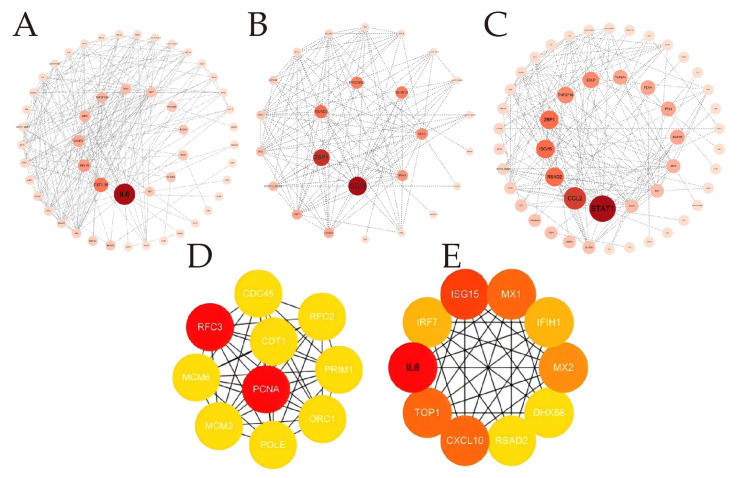
**Protein–protein interaction analysis.** (**A**–**C**) The analysis used the CytoNCA plugin to calculate the Betweenness Centrality (BC) index in the protein–protein interaction network based on STRING database. This helped identify DEGs related to the antiviral response during early BTV-1 infection. Divided into three groups: specific to 8 hpi (**A**), shared between 8 and 12 hpi (**B**), and specific to 12 hpi (**c**). (**D,E**) Top 10 genes identified PPI network by CytoHubba plug in 4 algorithms. From left to right are presented the 8 hpi group, and the 12 hpi group. Node circle size and color intensity are positively correlated with BC values; larger and darker circles indicate higher values.

Based on the aforementioned analysis, we performed PPI network analysis using Cytoscape software to identify the key hub genes involved in the antiviral response during the early phase of BTV-1 infection in OAEC II cells. Hub genes (such as CCL2, ISG15, MX1, ITGA5, IRF7 and RSAD2, etc.) are mainly closely related to antiviral defense mechanisms and immune responses (Figure 5).

### 3.6. Validation of Dif-mRNAs Using RT-qPCR

**Figure 6:**
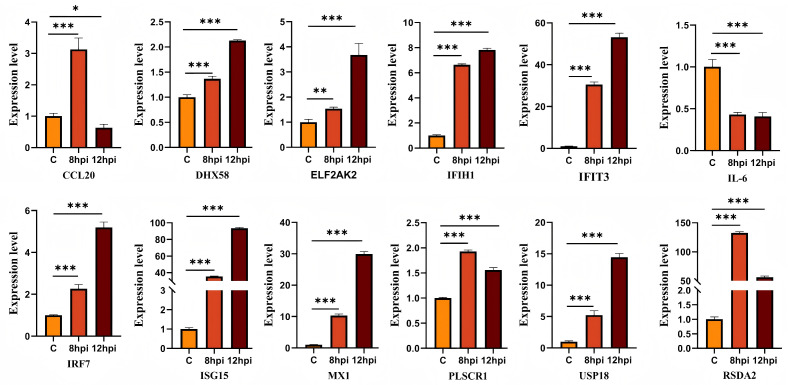
**Verification the expressions of DEGs.** ns indicates no significant difference (*p* > 0.05), * denotes *p* < 0.05, ** denotes *p* < 0.01, and *** denotes *p* < 0.001.

To validate the expression of dif-mRNAs, RT-qPCR analysis was performed for selected DEGs, including ISG-15, DHX58, PLSCR1, IRF7, IFIT3, USP18, IFIH1, and RSAD2 (Figure 6). The RT-qPCR results were consistent with the expression patterns observed in RNA-seq data, thus validating the reliability of the transcriptomic analysis.

## 4. Discussion

Decades of in-depth studies have unequivocally shown that BTV invades PMECs via the pulmonary capillary microvasculature, which caused profound cytotoxicity and vascular barrier dysfunction [9]. As the primary lung endothelial barrier against viral invasion, PMECs undergo dynamic pathophysiological remodeling post-infection, including disruption of endothelial junctional complexes and cytoskeletal rearrangement. This remodeling compromises endothelial barrier integrity, enabling BTV to transcytoses across the endothelium and subsequently infect AECs, thereby establishing a critical viral dissemination pathway.

### 4.1. Innate Immune Response and Interferon-Stimulated Gene Functions

The innate immune system acts as the first line of defense against viral infections by precisely recognizing pathogen-associated molecular patterns (PAMPs) through pattern recognition receptors (PRRs) [28]. This study revealed that BTV-1 infection significantly upregulated nucleic acid-sensing-related genes in OAECIIs at 8 and 12 hpi, including dsRNA sensors (MDA5/IFIH1, LGP2/DHX58, OAS2, OASL, and EIF2AK2), DNA sensors (cGAS and ZBP1), and non-canonical RNA sensors (ZNFX1 and PARP9) (Figure 5 and Table S5). The RIG-I-like receptor pathway is specifically involved in BTV infection, with DEGs at both time points exhibiting significant enrichment in this pathway (Figure 3E,F).

IFIH1 and DHX58 encode MDA5 and LGP2, respectively, which belong to the RIG-I-like receptor (RLR) family and signal through the MAVS adaptor protein. Their expression profiles showed consistent patterns across BTV-8-infected A549, BTV-1-infected OA3.Ts, and SARS-CoV-2-infected AECs [5,29,30]. LGP2 (DHX58), deficient in CARD domain, cannot directly transmit downstream signals. However, studies have shown that DHX58 potentiates IFIH1-mediated viral RNA recognition efficacy, consequently enhancing RLR-dependent IFN induction and amplifying antiviral signaling cascades [31]. Unlike RLRs, OAS-like receptors (OLRs) mediate antiviral responses by cleaving viral or host RNA upon dsRNA recognition. The dsRNA fragments generated via the OAS-RNaseL pathway can subsequently activate and amplifying signal transduction through the RIG-I-like receptor pathway [32,33]. PKR, another member of PRRs family encoded by the EIF2AK2 gene, recognizes viral dsRNA leading to phosphorylates eIF2α and inhibit viral protein synthesis [34]. This study demonstrates that OLR-related molecules and EIF2AK2 up-regulate expression to promote antiviral responses in early stage of BTV infection. However, reovirus S4 protein antagonizes OAS and PKR activation [35]. It remains unclear which protein to play this role in BTV.

Viral infection triggers cytoplasmic DNA accumulation, with demonstrated crosstalk between RNA and DNA sensing pathways [36]. BTV degrades cGAS via an NS3-protein-mediated autophagic mechanism, which inhibit the host cell’s DNA-sensing antiviral response by cGAS-STING pathway [37]. Our study revealed significant cGAS upregulation, which triggers a robust host antiviral defense through activation of the cytosolic DNA-sensing pathway at 8 and 12 hpi. This conclusion was drawn based on the analysis of enrichment of the cytosolic DNA-sensing pathway and IPA-predicted downstream regulatory networks (Figure 3E,F and Figure 4D). However, the results show that BTV suppresses cGAS expression after 12 hpi [37]. Prior to cGAS discovery, ZBP1 (DAI) was the first identified potential cytosolic DNA sensor [38]. Recent studies show ZBP1 also recognize RNA viruses and mediates both RIPK3-MLKL-dependentd necroptosis and NF-κB-driven antiviral responses [39,40]. Notably, the NS5 protein encoded by BTV S10-ORF2 exhibits structural homology with the Z-α domain-containing protein family, including ZBP1 [41].

The hypothesis that BTV NS5 competitively binds to viral Z-RNA, thereby preventing ZBP1 recognition and evading immune detection warrants experimental validation. Beyond known PRRs, this study reveals novel nucleic acid sensors ZNFX1, PARP9 and its binding partner DTX3L. Research has established that ZNFX1 binds viral RNA at mitochondria [42], whereas PARP9 recognizes RNA in a MAVS-independent manner [43]. The precise molecular mechanisms underlying how ZNFX1, PARP9, and DTX3L contribute to BTV recognition remain poorly understood and demand further elucidation. Understanding how host cells recognize viruses is critical for their role in antiviral immunity and may uncover viral escape strategies.

Upon recognition by RLRs or cGAS, viral nucleic acids activate interferon regulatory factors IRF3 and IRF7 [44], while ZBP1-mediated recognition induces IRF1 activation [45] to drive type I IFN signaling pathway gene transcription of host cells which leads to downregulation of BTV-encoded proteins, including structural components VP5 and VP7, as well as nonstructural proteins NS1 and NS3 [46]. Furthermore, the released IFN subsequently binds to IFNAR1/2 receptors on adjacent cells, initiating JAK-STAT signaling pathway that phosphorylates STAT1 and STAT2. The Phosphorylated STATs recruit IRF9 to form the ISGF3 complex, which translocates to the nucleus and binds to ISREs, thereby upregulating the transcription of ISGs and inhibiting viral replication [47]. Our data revealed dramatically upregulation of IFN-β, IRF7, IRF9, and STAT1 (Table S5).

Transcriptome analysis identified ISG15 as the predominant regulatory factor, with coordinated upregulation of USP18 and HERC5 (Figure 5 and Figure 6 and Table S5). In addition to these ISGs, some cytokines were shown to mediate their effects via ISG15 modification. Of particular significance, PCNA emerged as a pivotal gene at 12 hpi (Figure 5E), fulfilling a crucial role in preventing excessive mutation during DNA damage repair, where its ISGylation is indispensable for starting mismatch repair [48].

Research has established that Mx proteins, as a crucial ISGs, display specialized antiviral activities in early stage of virus infection. Specifically, MX1 exerts its antiviral activity by directly intercepting viral nucleocapsids prior to the initiation of viral genome replication [49] whereas MX2 exhibits potent antiretroviral specificity [50]. Our findings identify MX1 and MX2 as central hub genes in BTV infection at 8 hpi (Figure 5D), though their specific roles in mediating BTV-host interactions and antiviral responses remain to be fully elucidated. RSAD2 (Viperin), induced through activation of the JAK-STAT and IRF3/IRF1-mediated signaling pathway, exhibits potent antiviral activity against dengue virus, an arthropod-borne RNA virus [51]. BST-2 (Tetherin), a viral budding inhibitor functionally analogous to viperin, occurs as duplicated paralogs (BST-2A/B) in ovine and exhibits specific antiviral efficacy against retroviral infections [52]. Our results demonstrate notable expression of both Viperin and Tetherin (Table S5), indicating their possible involvement against BTV infection. PLSCR1 functions as an IFNγ-inducible autocrine factor that suppress COVID-19 infection and restricting SARS-CoV-2. Notably, even at basal endogenous levels, PLSCR1 alone is sufficient to restrict viral replication, and this activity is independent of IFN signaling pathways [53]. However, the precise molecular function and targets of PLSCR1 in BTV infection remain to be elucidated. Fortunately, our findings identified additional ISGs (RTP4, LY6E, IFIT3, XAF1, IFITM3, and MOV10) (Table S5), which may suggest their potential roles in antiviral defense.

### 4.2. Inflammation Response

Inflammation serves as a characteristic host defense mechanism against viral infection, dependent on the production and secretion of cytokines and chemokines by immune cells. In this study, DEG analysis demonstrated significant enrichment of the NOD-like receptor signaling pathway, cytokine-cytokine receptor interaction, and TNF signaling pathway at both 8 hpi and 12 hpi post-BTV infection (Figure 3E,F). Our results reveal a biphasic immunoregulatory response during early BTV infection, featuring coordinated downregulation of inflammatory mediators (IL-6, PYCARD, PTX3, SELP) alongside upregulation of immunomodulatory cytokines (IL-15, CXCL10, CCL2) (Table S5). This phenomenon indicates precise host control of inflammatory responses to maintain immune homeostasis during initial viral exposure. Given that dengue virus (DENV), another arthropod-borne virus, regulates IL-6 secretion in endothelial cells to facilitate its own replication [54,55,56], and that endothelial cells are also primary targets of bluetongue virus (BTV), BTV-1 may similarly exploit cytokine-mediated mechanisms to modulate IL-6 expression. To date, no study has directly demonstrated the effect of BTV replication on IL-6 transcription. Current evidence primarily highlights BTV’s antagonism of the type I interferon pathway, mediated through suppression of the JAK/STAT pathway, which disrupts interferon signaling [37,57]. Since both IL-6 expression and signal transduction depend on the JAK/STAT pathway [58,59], BTV-1 may indirectly suppress IL-6 transcription through this shared signaling cascade, thereby contributing to the downregulation of inflammatory mediators during early infection and helping to maintain host immune homeostasis. However, studies have demonstrated that as BTV infection progresses, the expression levels of CXC chemokine family members (e.g., CXCL8 and CXCL10) and the key inflammasome component NLRP3 significantly increase at 12 hpi (24 hpi, 36 hpi), potentially triggering an inflammatory storm [5,60]. The inflammatory storm induced by BTV infection further results in elevating microvascular permeability, thereby exacerbating tissue damage.

PTX3 not only acts as a critical regulator in the inflammasome pathway, but also through interaction with P-selectin (SELP), inhibits the initial adhesion of white blood cells to endothelial cells (i.e., leukocyte rolling), which reduces neutrophil recruitment at inflammatory sites to mitigates lung injury. Administration of PTX3 in acute lung injury models has been shown to markedly suppress inflammation and restore lung function [61]. These findings suggest that PTX3 may serve as a promising therapeutic target for alleviating the excessive inflammatory response induced by BTV infection. The chemokine CCL2 also plays a critical role in pulmonary inflammation initiation and progression. Various viral pathogens, such as HCMV, HIV, and coronaviruses, enhance CCL2 expression via host cell activation, generating a microenvironment conducive to viral replication. Recent studies reveal that CCL2 overexpression establishes a pathogenic virus-inflammation feedback loop, wherein infection-induced CCL2 responses promote further viral invasive infection [62,63]. In the context of BTV infection, we hypothesize that viral activation of NF-κB and MAPK signaling pathways stimulates CCL2 secretion, resulting in enhanced recruitment of monocytes/macrophages migration towards the inflamed area. This intricate interplay between PTX3, SELP, CCL2, and BTV-mediated inflammatory responses represents a critical area for further mechanistic investigation to clarify their coordinated roles in viral pathogenesis.

### 4.3. Disruption of Cellular Structural Integrity

Aquaporins (AQPs) are membrane proteins that regulate water and small molecule transport. AQP1 and AQP9 are two major subtypes responsible for water transport [64]. AQP1 also induces cell cycle arrest, senescence-associated secretory phenotype (SASP), DNA damage response, and angiogenesis regulation [65]. AQP9 is primarily expressed in hepatocytes and leukocytes, linking it to inflammation and infection [66]. In lungs, AQP9 regulates inflammation by promoting immune cell migration/activation and mitigating excessive responses to maintain homeostasis [67]. BTV infection markedly inhibits expression of critical channel proteins—including aquaporins (AQP1/AQP9) and potassium channel KCNK3—in OAECIIs despite their essential role in alveolar fluid homeostasis (Table S5). This phenomenon suggests that aquaporins contribute to early-stage inflammatory modulation, while their sustained downregulation may underlie pathogenesis of late-stage pulmonary edema. The exact mechanistic involvement of these channels in BTV-induced lung injury remains to be fully elucidated. The apical junctional complex (AJC), a critical intercellular structure composed of tight junctions (TJs) and adherens junctions, maintains essential barrier function through its structural integrity [68,69]. Multiple viruses have evolved distinct strategies to disrupt TJ integrity by either modulating AJC protein expression or facilitating their degradation, thereby compromising host barrier function [70]. BTV-infected OAECIIs significantly downregulates key AJC proteins (e.g., CLDN10/CLDN2), leading to TJ dysfunction and impaired host cell antiviral immunity. This phenomenon parallels that observed in other non-enveloped viruses, such as human respiratory syncytial virus (RSV) and human bocavirus (HBoV), which also exhibit similar intracellular mechanisms [71]. The mechanism underlying early-phase BTV-induced OAECIIs TJ disruption whether driven by host antiviral responses or viral evasion strategies requires further elucidation.

## 5. Conclusions

**Figure 7:**
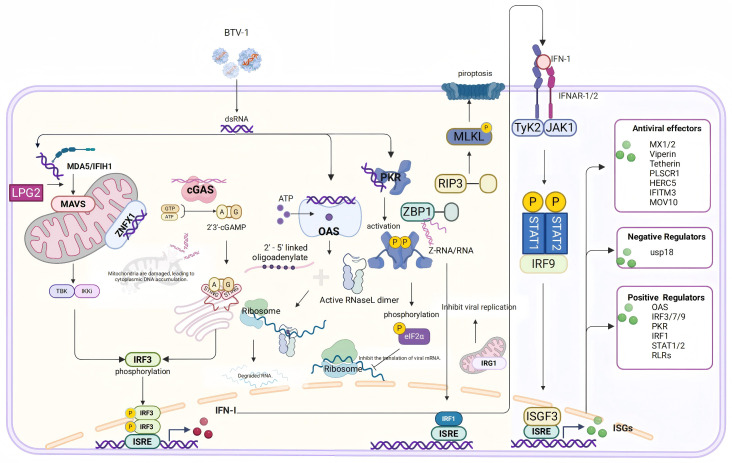
**Schematic representation of cytoplasmic nucleic acid pattern recognition and ISG activation following BTV infection.** Upon BTV infection, cytoplasmic nucleic acid sensors, such as MDA5 and cGAS, detect viral components, thereby triggering the expression and secretion of type I interferons. This subsequently enhances the expression of ISGs via the JAK-STAT signaling pathway. Furthermore, BTV can also be recognized by OAS, leading to direct nucleic acid degradation; by PKR, resulting in the inhibition of viral protein translation; and by ZBP1, which induces pyroptosis. Additionally, ZBP1 suppresses viral replication through the activation of IRF1.

This study applied RNA sequencing to analyze DEGs in OAECIIs during BTV-1 infection, uncovering transcriptional changes associated with innate immunity, inflammatory responses, and cell structural integrity. Integrated analysis revealed that MAD5, ZNFX1, cGAS, OAS, PKR, and ZBP1 are involved in viral recognition, while IFN-β, MX1/2, RSAD2, and PLSCR1 drive the early antiviral response. Furthermore, early BTV infection altered the expression of key inflammatory mediators—including PYCARD, PTX3, and SELP—and disrupted the expression of AQP1/9 and tight junctions (TJs), indicating compromised cellular architecture. These findings enhance our understanding of the host’s early immune defense against BTV-1, virus–host interactions, and the molecular mechanisms underlying bluetongue disease pathogenesis, offering a rational foundation for developing antiviral strategies (Figure 7). However, because the analysis was restricted to OAECIIs without comparative transcriptomic profiling in goat and sheep lung epithelial cells, the basis for species-specific differences in lung pathology—mild injury in goats versus severe damage in sheep—remains unexplained. Moreover, the lack of functional perturbation experiments limits causal inference regarding the roles of these pathways in viral infection, replication, and transmission.

## Figures and Tables

**Table 1:** Real-time fluorescent quantitative PCR primers.

GenBank	Target Genes	Full Name of Gene		Primer Sequences (5′-3′)
NM_007393.5	β-Actin		Forward	CCACTGTCGAGTCGCGTCC
			Reverse	ATTCCCACCATCACACCCTGG
XM_004004655	IFIH1	Interferon Induced with Helicase C Domain 1	Forward	GGTCAGCACGAGGAATAA
			Reverse	CTGTGGTAGCGATAAGCA
NC_056056.1	RSAD2	radical S-adenosyl methionine domain containing 2	Forward	GGTCTGCTGATGCTGAAGGAA
			Reverse	TCACAGGAGATGGCGAGGAT
XM_027960293	IFIT3	Interferon Induced Protein with Tetratricopeptide Repeats 3	Forward	GGCGGCTGAATGCTATGAGA
			Reverse	CTTCATGCTCAGTTGCTGGC
NC_056055.1	CCL20	C-C motif chemokine ligand 20	Forward	TGCTCTTGCTCCACCTCTG
			Reverse	GCTTGCTTCACCCACTTCTTC
NC_056065.1	ISG15	Ovis aries ISG15 ubiquitin like modifier	Forward	AGACTGTGGCTGTGCTCAAG
			Reverse	GCGGGTGCTCATCATCCAT
NC_056074.1	IRF7	interferon regulatory factor 7	Forward	CCGCACTACACCATCTACCTG
			Reverse	AGCCTGTTCCACCTCCATCA
NC_056054.1	MXI	Ovis aries MX dynamin like GTPase 1	Forward	TCGGTATCGTGGCAGAGAGT
			Reverse	TGGCAGTTCGGTGGAGGTT
NC_056054.1	PLSCR1	phospholipid scramblase 1	Forward	TGAGAGGCGAGAGGATGTACT
			Reverse	ATGGGAACTGGATGCCAAAGT
NC_056064.1	DHX58	DExH-box helicase 58	Forward	CGAAACTGGAGGTGCTGGAA
			Reverse	TCTGAGTCTTCTGGCTGTTGT
NC_056057.1	IL-6	Ovis aries interleukin 6	Forward	GGTTCAATCAGGCGATTTGCT
			Reverse	GTGTGTGGCTGGAGTGGTTAT
NC_056056.1	USP18	ubl carboxyl-terminal hydrolase 18	Forward	TGAGGAGCAGAGGAAGAGTGT
			Reverse	TTCAAGCGGATGGTGTAGAGG
NC_056056.1	eIF2aK2	eukaryotic translation initiation factor 2 alpha kinase 2	Forward	AGAAGGTAGAGCGTGAAGTGA
			Reverse	TCGTCAATCCATTCCGCCAAT
KM099648.1	NS4	non-structural protein NS4	Forward	ATGGTGAGGGGGCACAACAGAA
			Reverse	CCCATCCTCCTCTGCTCGCT

**Table 2:** IPA analysis identified upstream regulators and downstream effects of DEGs in BTV-1 infection at 8 hpi.

Upstream Regulator	Molecule	Activation Z-Score	*p*-Value of Overlap	Target Molecules Number
TNF	Cytokine	2.051	7.54 × 10^−13^	90
IMMUNOGLOBULIN(complex)	complex	1.960	3.01 × 10^−12^	71
IRF7	transcription regulator	3.296	2.43 × 10^−11^	18
tetradecanoylphorbol acetate	chemical drug	1.737	5.82 × 10^−11^	60
dexamethasone	chemical drug	−0.893	2.19 × 10^−10^	91
vidutolimod	chemical drug	2.673	5.35 × 10^−10^	10
IFNAR2	transcription regulator	2.000	8.42 × 10^−10^	15
SN-011	chemical reagent	−1.722	1.24 × 10^−9^	13
pyridostatin	chemical reagent	3.426	1.25 × 10^−9^	12
STAT1	transcription regulator	0.968	1.73 × 10^−9^	20
TNF(family)	group	−0.458	2.54 × 10^−9^	23
MAPK1	kinase	−2.848	2.55 × 10^−9^	31
TWISTI	transcription regulator	0.600	2.84 × 10^−9^	24
TREX1	enzyme	−2.492	3.27 × 10^−9^	18
MAP3K7	kinase	0.015	3.72 × 10^−9^	17
TGFβ1	growth factor	0.367	3.91 × 10^−9^	82
NONO	transcription regulator	2.646	5.70 × 10^−9^	14
IL1B	Cytokine	1.496	6.83 × 10^−9^	56
CHROMR	other	2.755	7.59 × 10^−9^	17
WNT3A	Cytokine	0.362	9.29 × 10^−9^	26
INTERFERON ALPHA(family)	group	2.376	1.00 × 10^−8^	35
Tretinoin	biological drug	2.615	1.08 × 10^−8^	70
Lipopolysaccharide	Cytokine	1.953	1.09 × 10^−8^	96
F3	transcription regulator	−3.267	1.64 × 10^−8^	19
IFNL1	Cytokine	2.436	1.87 × 10^−8^	11
IFN BETA(family)	group	2.507	2.73 × 10^−8^	21
Stallimycin	biological drug	1.632	2.91 × 10^−8^	10
IL17A	Cytokine	−0.695	4.17 × 10^−8^	28
IFNAR1	transcription regulator	2.741	4.42 × 10^−8^	17
IRF3	transcription regulator	1.896	4.28 × 10^−8^	33

**Table 3:** IPA analysis identified upstream regulators and downstream effects of DEGs in BTV-1 infection at 12 hpi.

Upstream Regulator	Molecule	Activation Z-Score	*p*-Value of Overlap	Target Molecules Number
TNF	cytokine	1.43	1.2 × 10^−13^	87
pyridostain	chemical reagent	3.713	1.27 × 10^−12^	14
TGFB1	growth factor	−0.474	8.31 × 10^−12^	84
NONO	transcription regulator	2.97	1.41 × 10^−11^	16
TWISTI	transcription regulator	0.187	1.64 × 10^−11^	26
etrogen	chemical drug	1.043	2.64 × 10^−11^	29
ETV6	transcription regulator	−2.731	2.73 × 10^−11^	13
TP53	transcription regulator	−1.279	3.30 × 10^−11^	87
PGR	ligand-dependent nuclear	−0.422	5.00 × 10^−11^	36
ETV3	transcription regulator	−3.317	5.65 × 10^−11^	11
TNIK	kinase	3.286	5.65 × 10^−11^	11
F3	transcription regulator	−3.946	9.37 × 10^−11^	21
vidutolimod	chemical drug	3.357	1.80 × 10^−10^	15
STAT1	transcription regulator	1.858	2.62 × 10^−10^	30
IRF7	transcription regulator	3.910	7.28 × 10^−10^	16
IL18	Cytokine	1.104	2.85 × 10^−9^	54
dexamethasone	chemical drug	−2.278	3.19 × 10^−9^	83
IFNA2	cytokine	3.616	6.93 × 10^−9^	21
CG(complex)	complex	−1.786	6.97 × 10^−9^	34
CSF1	cytokine	0.373	9.13 × 10^−9^	27
IFNAR(family)	group	3.676	9.35 × 10^−9^	14
IRGM	enzyme	−2.949	9.54 × 10^−9^	12
IFNAR1	transcription regulator	3.539	1.34 × 10^−8^	17
NTRK1	kinase	−1.666	1.38 × 10^−8^	23
IRF1	transcription regulator	2.842	1.94 × 10^−8^	19
IL6	Cytokine	0.321	2.10 × 10^−8^	41
doxoorubicin	chemical drug	0.260	2.26 × 10^−8^	41
FGF2	growth factor	−0.402	2.29 × 10^−8^	32
RIPK2	kinase	2.376	3.61 × 10^−8^	15
CGAS	enzyme	2.250	3.68 × 10^−8^	12

## Data Availability

The original contributions presented in this study are included in the article/Supplementary Material. Further inquiries can be directed to the corresponding author(s).

## Supplementary Materials

The following supporting information can be downloaded at: https://www.mdpi.com/article/10.3390/ani16020243/s1, Table S1: GO enrichment analysis of differentially expressed transcripts between the 8 hpi group and the control group; Table S2: GO enrichment analysis of differentially expressed transcripts between the 12 hpi group and the control group. Table S3: KEGG enrichment analysis of differentially expressed transcripts between the 8 hpi group and the control group. Table S4: KEGG enrichment analysis of differentially expressed transcripts between the 12 hpi group and the control group. Table S5: There is a gene expression profile analysis table for each group.
